# Spatial and temporal variability of event runoff characteristics in a small agricultural catchment

**DOI:** 10.1080/02626667.2020.1798451

**Published:** 2020-08-05

**Authors:** Xiaofei Chen, Juraj Parajka, Borbála Széles, Peter Strauss, Günter Blöschl

**Affiliations:** a Centre for Water Resource Systems, TU Wien, Vienna, Austria; b Institute of Hydraulic Engineering and Water Resources Management, TU Wien, Vienna, Austria; c Federal Agency for Water Management, Institute for Land and Water Management Research, Petzenkirchen, Austria

**Keywords:** event runoff coefficient, recession time constant, runoff peak, small agricultural catchment, runoff generation

## Abstract

The objective of this study is to investigate the factors that control event runoff characteristics at the small catchment scale. The study area is the Hydrological Open Air Laboratory, Lower Austria. Event runoff coefficient (Rc), recession time constant (Tc) and peak discharge (Qp) are estimated from hourly discharge and precipitation data for 298 events in the period 2013–2015. The results show that the Rc and their variability tend to be largest for the tile drainages (mean Rc = 0.09) and the main outlet (mean Rc = 0.08) showing larger Rc in January/February and smaller Rc in July/August. Tc does not vary much between the systems and tends to be largest at the main outlet (mean Tc = 6.5 h) and smallest for the tile drainages (mean Tc = 4.5 h). Groundwater levels explain the temporal variability of Rc and Tc more than soil moisture or precipitation, suggesting a role of shallow flow paths.

## Introduction

Formation of runoff during rainfall events is controlled by climate and physiographic catchment characteristics and depends on the runoff generation processes. The infiltration excess mechanism is mainly controlled by precipitation intensity and infiltration capacity while the saturation excess mechanism is mainly controlled by precipitation volume and soil depth (Tian *et al*. 2012). Both affect event runoff characteristics such as the runoff coefficient, the recession time constant and the runoff peaks (Merz *et al*. 2006, Ruggenthaler *et al*. 2015).

Event runoff coefficient indicates the ratio of direct flow volume to total event rainfall, so it is an important parameter in engineering design (Blume *et al*. 2007, Viglione *et al*. 2009). It indirectly reflects not only hydrological conditions but also catchment characteristics and different runoff generation mechanisms. Especially in agricultural catchments, understanding factors controlling runoff coefficient is an essential information for management agricultural practices and preventing erosion (García-Ruíz *et al*. 2008). The runoff recession time constant is a measure of the time required after rainfall for streams to return to their base flow levels (Czikowsky and Fitzjarrald 2004). It is usually described by a simple linear reservoir model and indicates the interaction between groundwater and surface flow (Merz *et al*. 2006). Understanding the factors controlling recession flows is critical mainly for water supply, irrigation, water quality and erosion. Besides, the magnitude of event peak flow is an important hydrological characteristic used in flood risk and design estimation (Gottschalk and Weingartner 1998, La Torre Torres *et al*. 2011).

Previous studies examining event runoff characteristics found that the controlling factors differ with the spatial scale. The connectivity between the “infiltrating” and “runoff producing” areas explains the variability of event runoff characteristics from plot to small catchment scales, and as found by Joel *et al*. (2002) and Cerdan *et al*. (2004), the runoff response at this scale tends to significantly decrease with increasing catchment size. The differences in connectivity of flow paths can explain the differences in runoff response between plot and small catchment scale, but are less important for larger catchments. In small catchments, land use plays an important role (Cerdan *et al*. 2004) and may have a significant impact on streamflow recession by increasing recession constant with an increasing percentage of impervious areas (Burns *et al*. 2005) and affect the variability in frequency, seasonality and magnitude of flood peaks (García-Ruíz *et al*. 2008).

Evaluation of event runoff characteristics at the plot and hillslope scale based on experiments show that the main controls depend on the interactions between infiltration rate, change in soil water storage and drainage of the soil water (Scherrer *et al*. 2007, Ruggenthaler *et al*. 2015). At the catchment scale, runoff formation is less understood, mainly because of the large spatial variability of the environment and the connectivity of runoff flow paths (Western *et al*. 1998, Cerdan *et al*. 2004, James and Roulet 2007, Silasari *et al*. 2017). Statistical analyses of flow data in medium and large catchments show that the main controls of the spatial variability of event runoff characteristics at the regional scale are mean annual precipitation and the runoff regime (Merz *et al*. 2006), physiographic catchment characteristics (Gottschalk and Weingartner 1998) and antecedent soil moisture (Norbiato *et al*. 2009). Often, similarly to the plot scale (Ruggenthaler *et al*. 2015), only a weak correlation between event runoff characteristics and soil type or land use has been found at the regional scale (Merz *et al*. 2006).

Temporal changes in event runoff characteristics at the regional scale are mostly related to the volume of rainfall and pre-event soil moisture (La Torre Torres *et al*. 2011, Penna *et al*. 2011, Chifflard *et al*. 2018, Tarasova *et al*. 2018a, 2018b). The runoff coefficients tend to be high in the winter and spring when soil moisture is high and lower in the summer when catchments are dryer (Merz and Blöschl 2009). The role of pre-event soil moisture at the plot scale depends on the subsurface storage of the catchments and the dominant runoff generation processes (Scherrer *et al*. 2007, Rodríguez-Blanco *et al*. 2012). In regions with poorly developed soils, the relationship between runoff coefficients and pre-event soil moisture tends to be strongly nonlinear, while permeable soils tend to exhibit more linear relationships (Tarasova *et al*. 2018a, 2018b).

The results of previous studies on event runoff characteristics indicate that there is a scale gap between plot experiments and comparative regional analyses of medium to large catchments. The objective of the study is thus to investigate the spatial and temporal variability of event runoff characteristics at the small catchment scale. The aim is to identify and evaluate the factors that control the variability of the event runoff coefficient, the recession time constant and the peak discharge in a small agricultural catchment where the individual tributaries are characterized by different runoff generation systems. The investigation evaluates the role of event precipitation, soil moisture and groundwater for event runoff characteristics of the different runoff generation systems.

## Study area and data

**Figure 1.**
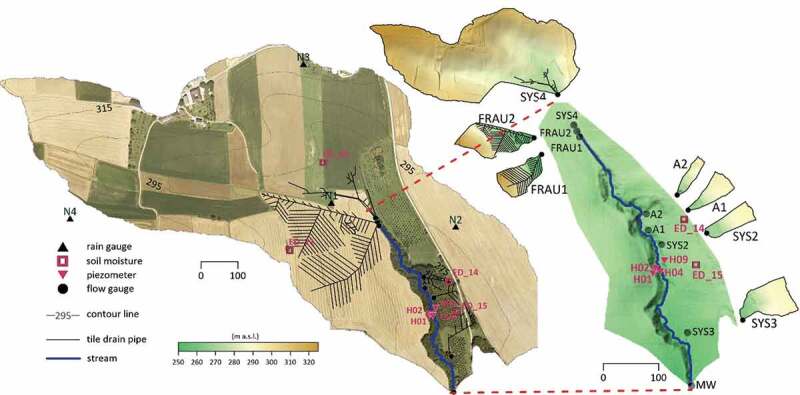
Study area and location of the rain gauges (black triangles), soil moisture sensors (squares), groundwater piezometers (red triangles) and stream gauges (circles) Left: ortho-photo of the HOAL catchment and location of the tile drainage system. Right: zoom in to the tributary catchments and their topography.

The study area is a small experimental catchment, the Hydrological Open Air Laboratory (HOAL) in Petzenkirchen, Lower Austria (Fig. 1). HOAL is an agricultural catchment situated approximately 100 km west of Vienna (48°9′N, 15°9′E). The main land use is arable land (87%), forest and pastures. The size of the catchment is 66 ha and the elevation varies between 268 and 323 m a.s.l. The mainstream is approximately 620 m long and has a medium slope of 2.4% (Blöschl *et al*. 2016).

The climate of the region is classified as warm and temperate (Cfb class of the Köppen-Geiger climate classification) with a mean annual temperature of 9.3°C, a mean annual precipitation of around 750 mm and a mean annual flow of about 4 L/s (Blöschl *et al*. 2016). The main geology classes of the HOAL consist of Tertiary fine sediments and fractured siltstone of the Molasse zone and the dominant soil types are Cambisols (57%), Kolluvisol (16%), Planosols (21%) and Gleysols (6%) (Blöschl *et al*. 2016).

The observation period analysed in this paper is 2013–2015. Rainfall is measured by four OTT Pluvio rain gauges within or near the catchment (Fig. 1). Streamflow is measured by calibrated H-flumes with pressure transducers (Fig. 1). Both measurements are carried out at 1 min temporal resolution. The gauged tributaries represent different runoff generation systems (Table 1). The contributions from the wetland areas in the south-eastern part of the catchment are measured at sites A1 and A2. While Sys2 and Sys3 are perennial streams and contribute to the flow of the mainstream throughout the whole year, Frau1, Frau2 are ephemeral and mainly consist of tile drains. During low flow conditions, Sys3 behaves as a combination of a tile drain and a wetland as it collects water from saturated areas near the stream. The upper part of the stream is piped and enters the mainstream at inlet Sys4. The catchment area and mean flow of each runoff generation system are given in Table 1.

**Table 1.** Characteristics of HOAL catchment and its sub-catchments used in this paper. SD: standard deviation.

Gauge	Runoff generation system	Estimated drainage area (ha)	Mean drainage area slope (%)	SD of mean drainage area slope	Forest coverage (ha)	Mean streamflow 2013–2015 (mm/h)	SD of mean streamflow	Soil moisture sensor station
A1	Wetland	2.1	8.90	6.56	0.25	0.025	0.036	ED14
A2	Wetland	1.1	11.53	6.55	0.17	0.029	0.024	ED14
FRAU1	Ephemeral tile drain	3.1	7.00	2.21	0.00	0.024	0.053	ED21
FRAU2	Ephemeral tile drain	4.8	9.07	3.74	0.01	0.012	0.028	ED21
SYS2	Natural drainage	2.4	9.73	7.05	0.45	0.026	0.018	ED15
SYS3	Natural drainage (with wetland)	4.3	10.04	6.06	0.61	0.008	0.015	ED15
SYS4	Natural drainage (inlet pipe)	37.4	10.91	5.84	1.73	0.007	0.011	ED22
MW	Outlet (aggregated system)	65.8	10.65	6.67	6.32	0.023	0.045	Mean of ED14, ED15, ED21 and ED22

All streamflow data were quality checked and aggregated to an hourly time step. Catchment boundaries were derived from a 1-m digital elevation model (DEM), additionally accounting for the position of the tile drain pipes (Széles *et al*. 2018).

For the analysis of initial soil moisture, the soil moisture measurements from sensors (ED14, ED15, ED21 and ED22) at 5, 10 and 20 cm depth were integrated over depth to represent the mean profile soil moisture. The soil moisture monitoring started in August 2013, so for the first 15 events, the initial soil moisture information is missing. The sensors used for different sub-catchments are shown in Table 1. For the analysis of the initial groundwater conditions, the mean value of four piezometer readings (H01, H02, H04 and H09) is calculated and used in all sub-catchments and the main outlet. The initial groundwater levels can slightly differ between the sub-catchments as the exact time of event start can be different.

## Methods

The rainfall–runoff events were separated by using the automatic method of Merz *et al*. (2006). It consists of estimating the catchment precipitation, determining direct runoff and baseflow, identifying the start and end of the events and calculating the event runoff characteristics, i.e. peak discharge, runoff coefficient and recession time constant. Hourly catchment precipitation was estimated based on measurements at four rain gauges by the Thiessen polygon method. The event runoff coefficient relates direct flow volume to total event rainfall, so it was necessary to separate direct quickflow and baseflow. Direct quickflow runoff arises from rainfall that contributes immediately to streamflow during an event, while baseflow contributes to streamflow with a significant delay. Baseflow and direct runoff contributions were determined by the Chapman and Maxwell (1996) digital filter. If the ratio of direct runoff and baseflow at time *t* was larger than a threshold value of parameter qdrat and there was no larger flow in the previous and following imax hours, the flow at time *t* was considered as a peak. The parameters qdrat and imax were set to 2 and 12 h, respectively, based on sensitivity analyses (not shown here), consistent with the parameters of Merz *et al*. (2006) for Austria. For each peak, the start of the event was searched backwards to find the time when the direct runoff is less than 1% of the direct runoff at the time of the peak. The number of time steps in the backward search (size of the time window) depends on the characteristic timescale of an event (Merz *et al*. 2006). If no such point in predefined time window is found, a higher limit for minimum direct runoff is allowed (stepwise increased from 1% to 40%). The end time was found in an analogue way by searching forwards. The runoff coefficient Rc and recession time constant Tc were determined in two steps. In the first step, Rc and Tc values were automatically calibrated by using the shuffled complex evolution optimization scheme (Duan *et al*. 1992). The linear reservoir model was fitted to the direct flow by minimizing the root-mean-square difference between observed and simulated runoff. In the second step, final hydrographs were visually checked and, in some cases, Tc was manually adjusted and fixed to match the form one would separate manually. After fixing, Rc is again automatically optimized until the simulated hydrograph fits the observation. More details on the method are given in Merz *et al*. (2006).

**Figure 2.**
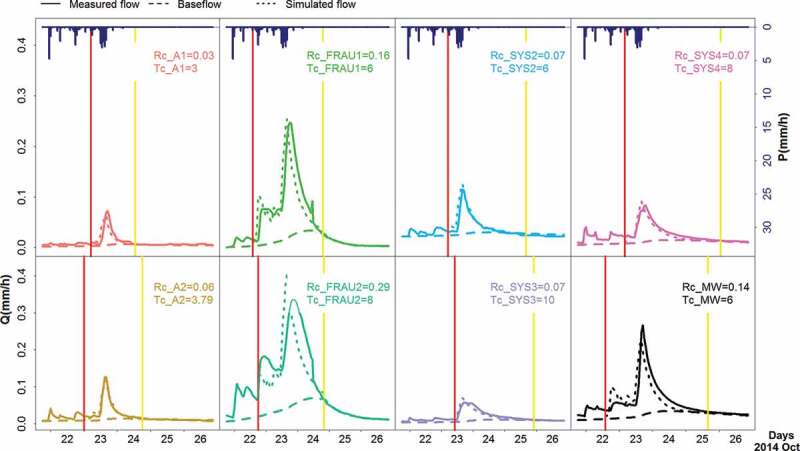
Example of observed and simulated runoff events at the main outlet (MW) and seven tributaries in HOAL catchment in October 2014. Runoff (dotted lines) is simulated by the linear reservoir model.

An example of an identified event in October 2014 is shown in Fig. 2. The runoff response to precipitation differs between the tributaries, but the linear reservoir model fits the observed streamflow well. For this event, the runoff peaks (in units mm/h) of the tile drain systems Frau 1 and Frau2 are noticeably larger than those of the wetlands A1 and A2.

**Figure 3.**
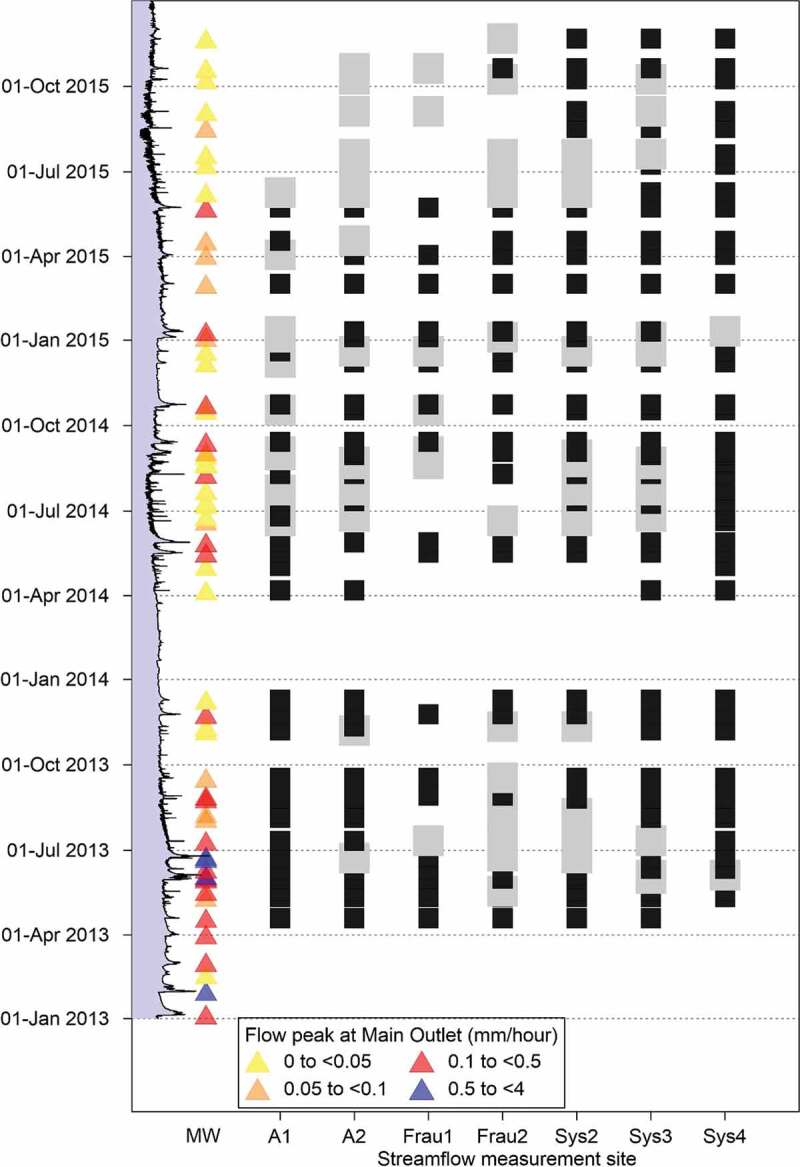
Flood peaks at the main outlet (triangles) in the period 2013–2015 and identified runoff events (black rectangles) at the tributaries. Grey symbols indicate available streamflow but no identified runoff event at the tributaries. In case of data gaps at the tributaries because of equipment failure or regular maintenance, no symbol is plotted. On the left side the hydrograph at the main outlet (MW) is presented.

In total, 57 runoff events were identified at the main outlet in the period 2013–2015. Figure 3 shows the time sequence of these events both at the main outlet (MW) and the seven tributaries (black squares). In case no event was identified at a tributary, but streamflow data were available, grey symbols are plotted. In total 298 event hydrographs were identified at the eight gauges in the HOAL, which are summarized in the Appendix (Table A1).

## Results

Table 2 presents a summary of the event runoff characteristics of the identified event hydrographs. The mean Rc of all hydrographs at the main outlet is less than 0.08 with a standard deviation (*σ*) of 0.09. The mean Rc of the tile drainage systems is somewhat larger (mean Rc is 0.09 and *σ* is 0.09), while those of the wetlands and natural drainage systems are notably smaller (mean Rc is 0.04 and 0.03, and *σ* is 0.03 and 0.02, respectively). While the largest mean recession time constant is found at the main outlet (mean Tc = 6.6 h, *σ* = 7.6 h), the smallest mean Tc is observed for the tile drainage systems (mean Tc = 4.2 h, *σ* = 2.5 h). The difference in mean Tc between the systems is generally smaller than the variability of Tc between different events at the same gauge. The relative magnitudes of the mean peak discharges (in mm/h) of the different systems are similar to those of the runoff coefficients, i.e. compared to wetland systems, mean peaks are larger for the tile drainage systems and smaller for the natural drainage system. At the main outlet, the mean peaks are the largest (mean Qp: 0.2 mm/h and *σ*: 0.4 mm/h).

**Table 2.** Summary of runoff event characteristics in the HOAL in the period 2013–2015. Minimum, maximum, mean and standard deviation (*σ*) of runoff coefficient (Rc), recession time constant (Tc) and peak discharges (Qp) evaluated for the different runoff generation systems (wetland, tile drainage, natural drainage and the main outlet MW). The statistical evaluation is based on the events listed in Table A1.

		Wetland		Tile drainage		Natural drainage			Outlet
Features of event		A1	A2	FRAU1	FRAU2	SYS2	SYS3	SYS4	MW
Rc	Min Max Mean *σ*	0.0030.0820.0380.021	0.0060.2220.0540.046	0.001 0.297 0.091 0.094	0.0003 0.386 0.086 0.095	0.006 0.089 0.036 0.028	0.001 0.094 0.022 0.022	0.003 0.096 0.021 0.022	0.004 0.334 0.077 0.093
Tc (h)	Min Max Mean *σ*	1.0017.05.373.80	0.50 21.9 5.41 5.13	0.50 10.0 4.22 2.49	1.00 15.0 5.65 3.33	1.00 17.0 5.76 3.77	0.50 25.0 5.44 4.78	1.00 25.0 4.61 5.28	0.10 32.6 6.57 7.58
Qp (mm/h)	Min Max Mean *σ*	0.0170.2210.0910.057	0.015 0.344 0.107 0.067	0.004 0.454 0.142 0.153	0.003 0.335 0.128 0.104	0.023 0.249 0.087 0.055	0.004 0.267 0.044 0.050	0.008 0.280 0.052 0.055	0.018 3.038 0.198 0.436

**Figure 4.**
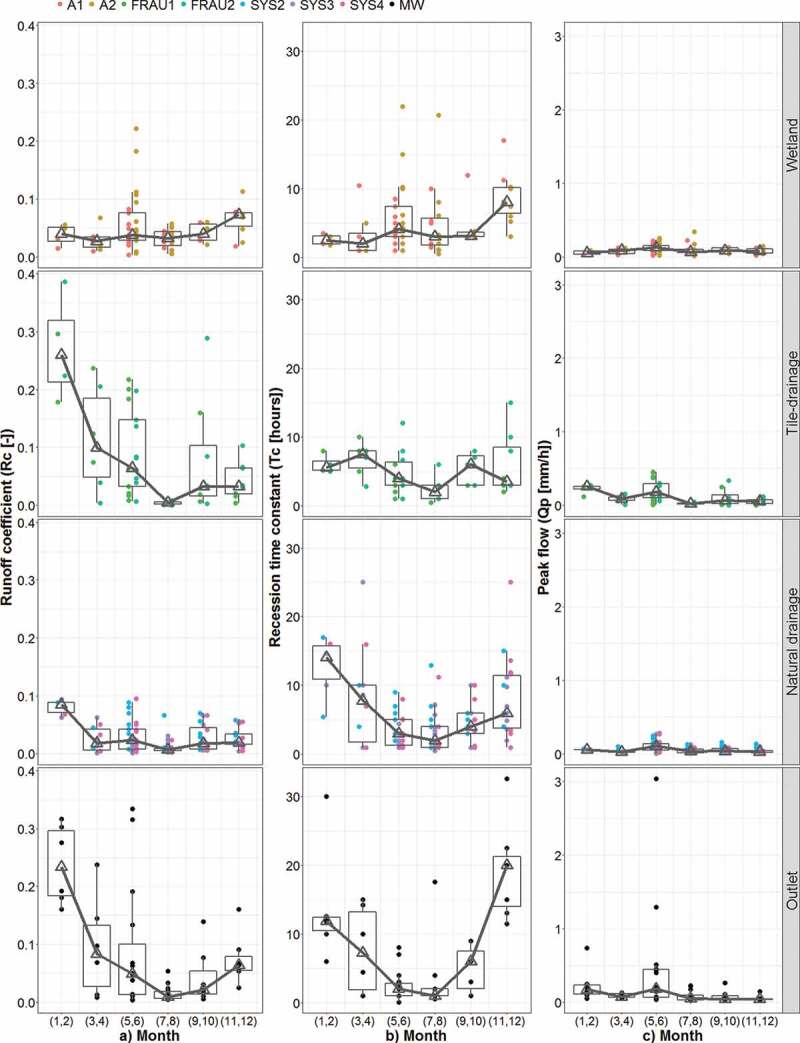
Seasonal variability of event runoff characteristics in the HOAL in the period 2013–2015: (a) runoff coefficient, Rc, (b) recession time constant, Tc, and (c) peak discharge, Qp, for four different runoff generation systems (wetland, tile drainage, natural drainage and main outlet). The boxes represent the 25% and 75% quantiles and the triangles the medians. The lower whisker represents the smallest observation greater than or equal to the 25% quantile – 1.5 × IQR and the upper whisker the largest observation less than or equal to the 75% quantile +1.5 × IQR, where IQR is the difference between the 25 and 75% quantiles. Each circle represents an event.

The seasonal variability of the event runoff characteristics is presented in Fig. 4. The results of individual events are grouped (for better visual appearance) into bi-monthly classes. The results show that seasonal variability of Rc differs between the systems (Fig. 4(a)). For the main outlet (MW) and the tile drain systems (Frau1 and Frau2) the largest runoff coefficients (median over 0.2) occur in January/February, while from July to October the median is below 0.035. In the wetlands, the runoff coefficients vary only slightly between months with a median between 0.03 and 0.07, and in May/June the scatter is largest. In the natural drainage systems (Sys2, Sys3 and Sys4) the runoff coefficients are largest in January/February (median about 0.08), but in the other months, they are rather small and similar to the wetlands. Overall, the runoff coefficients in the HOAL catchments are small, and the median Rc is less than 0.03. There are only five events with Rc larger than 0.3 in sub-catchments and the main outlet, and the largest runoff coefficient (Rc = 0.38) is observed in the ephemeral tile drain (Frau 2) in February 2015.

The seasonal variability of the recession time constant Tc (Fig. 4(b)) is the largest in the natural drainage systems and the main outlet. Tc in these two systems is particularly large in January/February when the median exceeds 10 h. The smallest Tc is observed in July/August and except wetlands, the median Tc is below 3 h. The wetlands have the smallest inter-seasonal variability, and large Tc is mainly observed in May/June and November/December. Interestingly, at MW, the largest Tc occurs in November/December, while the largest Rc occurs two months later in January/February.

The peak discharge does not vary much seasonally in HOAL catchments with the exception of a number of large events at the main outlet in June.

**Figure 5.**
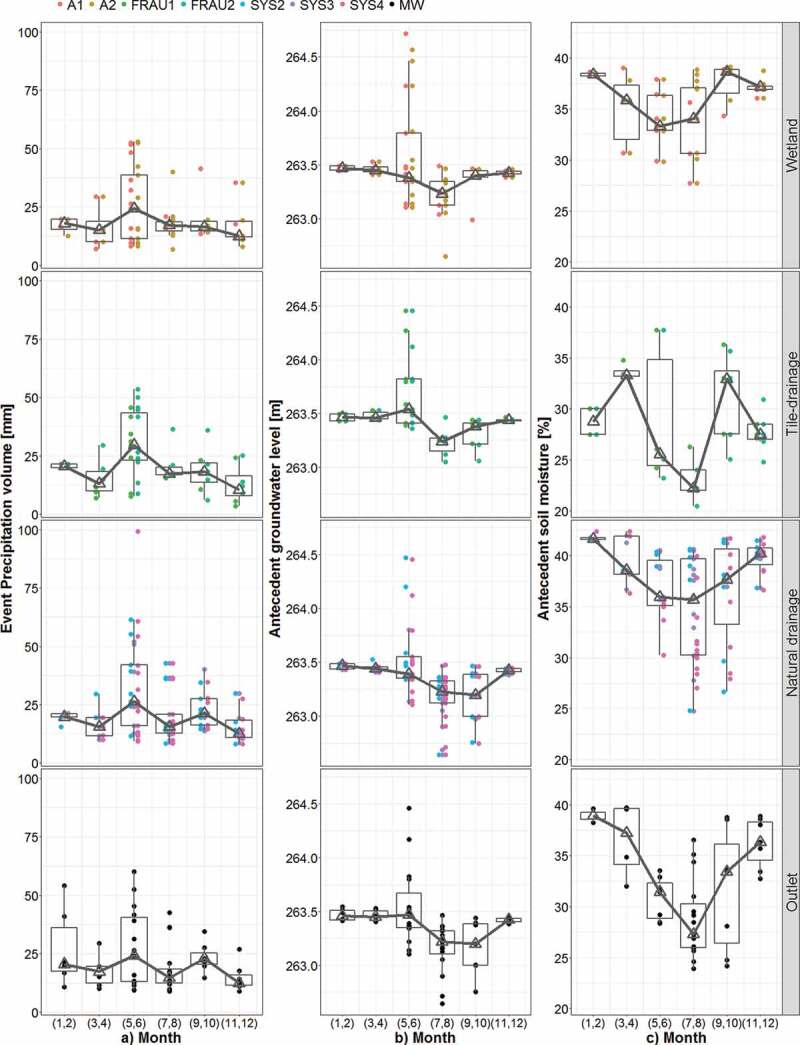
Seasonal variability of selected controls of event runoff characteristics in the HOAL in the period 2013–2015: (a) event precipitation volume (b) initial groundwater level and (c) initial soil moisture, for four different runoff generation systems (wetland, tile drainage, natural drainage and main outlet). For explanation of boxes see.Fig. 4

The seasonal variability of selected factors that may control event runoff generation is presented in Fig. 5. The largest precipitation volumes (Fig. 5 left panel) with a median larger than 25 mm/event occur in May/June. In these months, more than 25% of the events have precipitation volumes larger than 40 mm. Interestingly, the larger precipitation volumes in May/June are not generally reflected in higher groundwater or soil moisture levels (Fig. 5 middle and right panels) indicating drier subsurface conditions due to enhanced evaporation, and most of the runoff events are caused by convective rainfall that does not usually saturate the soils. As would be expected in the climate of the HOAL, soil moisture varies strongly seasonally with drier months from May to August. The seasonal variability of the groundwater levels is smaller.

**Figure 6.**
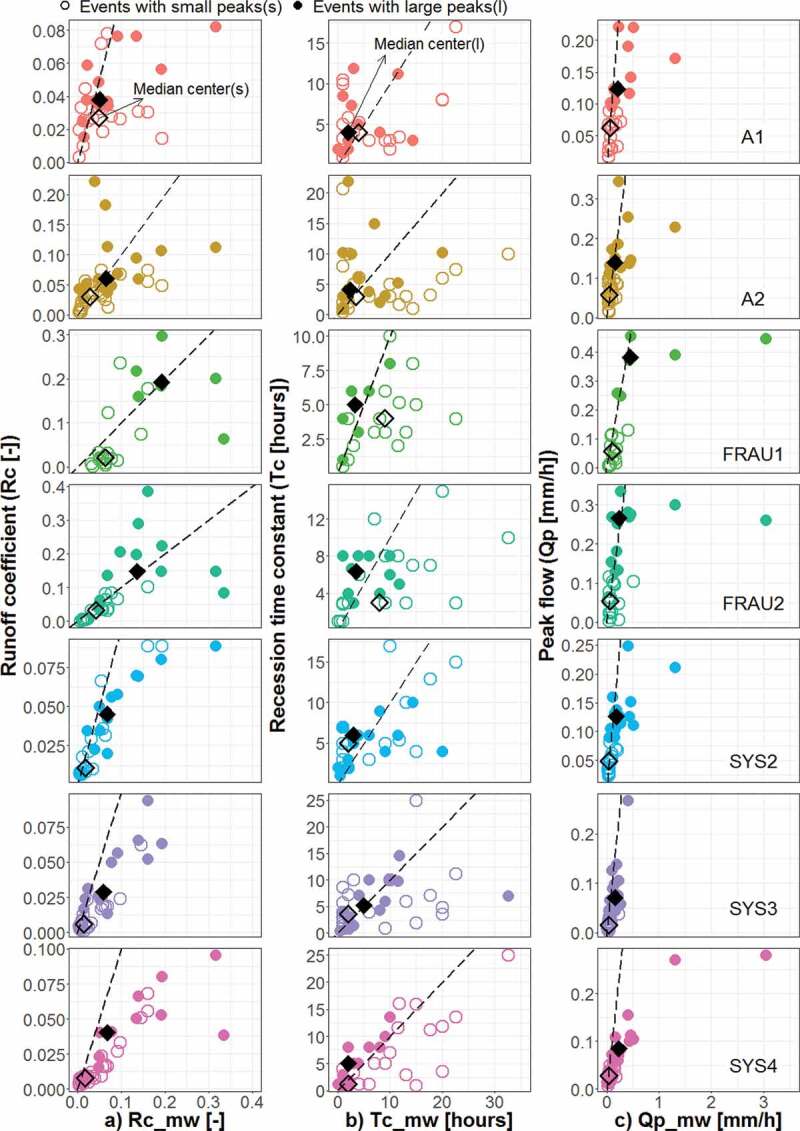
Event runoff characteristics of the tributaries plotted against those of the main outlet: (a) runoff coefficient, Rc, (b) recession time constant, Tc, and (c) peak discharge, Qp. Open and full circles indicate Qp at the tributaries that is, respectively, smaller and larger than the mean (0.09, 0.11, 0.14, 0.13, 0.09, 0.04 and 0.05 mm/h in tributaries A1, A2, FRAU1, FRAU2, SYS2, SYS3 and SYS4, respectively). Open and full diamonds indicate the median centres of the groups smaller and larger than that mean, respectively. The dashed line is the 1:1 line.

**Figure 7.**
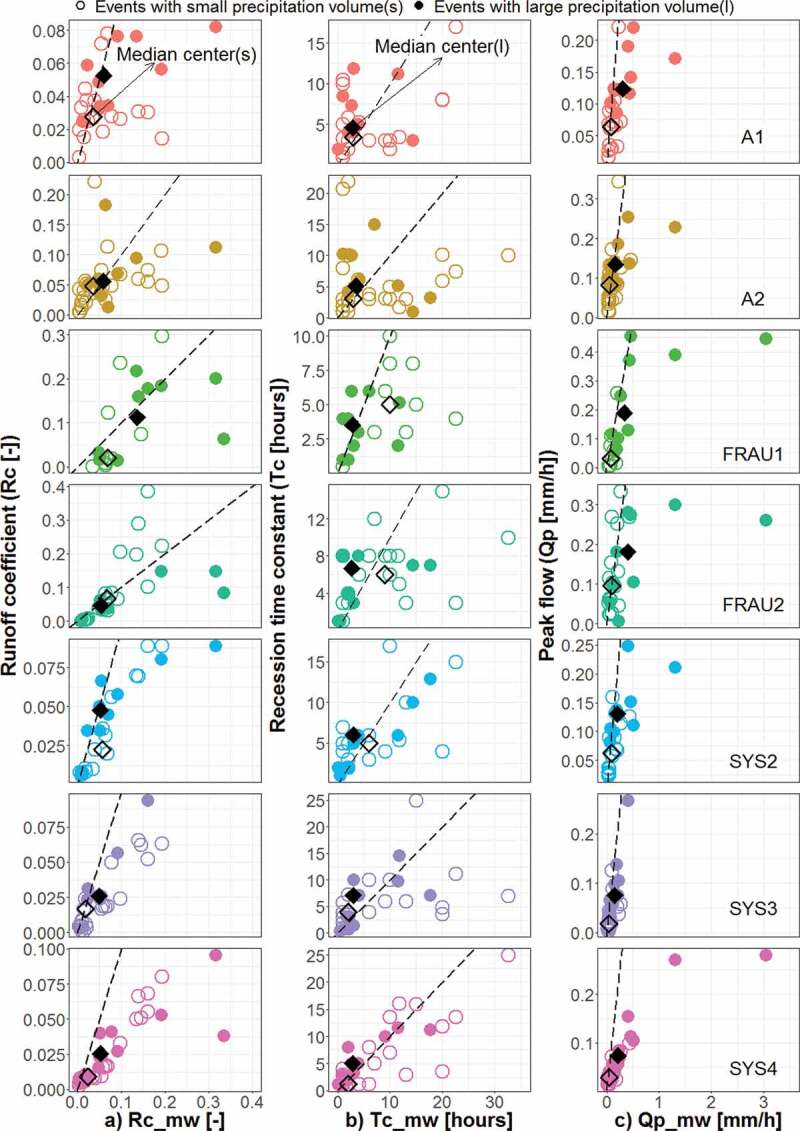
Event runoff characteristics of the tributaries plotted against those of the main outlet: (a) runoff coefficient, Rc, (b) recession time constant, Tc, and (c) peak discharge, Qp. Open and full circles indicate precipitation volume, Pvol, of the tributaries that is smaller and larger than the mean (22.3, 20.0, 20.9, 25.4, 24.3, 20.0 and 22.5 mm in tributaries A1, A2, FRAU1, FRAU2, SYS2, SYS3 and SYS4, respectively).

**Figure 8.**
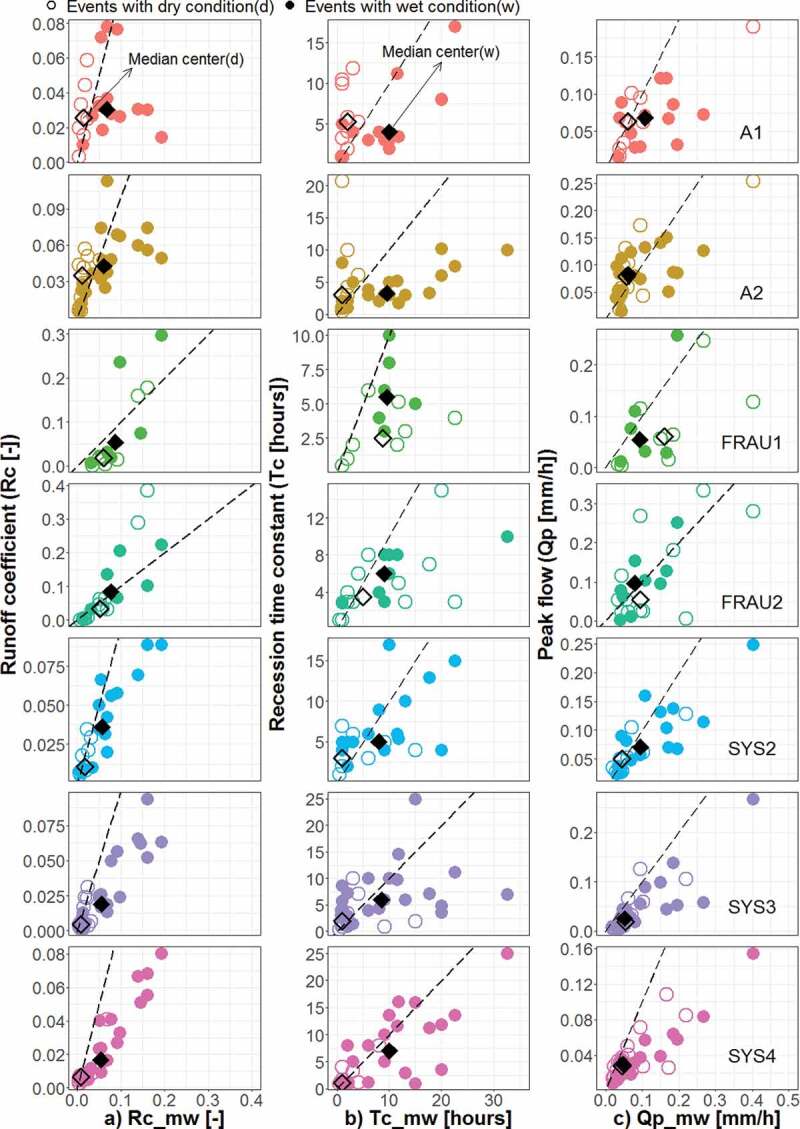
Event runoff characteristics of the tributaries plotted against those of the main outlet: (a) runoff coefficient, Rc, (b) recession time constant, Tc, and (c) peak discharge, Qp. Open and full circles indicate soil moisture prior to the event, PreSM, of the tributaries that is, respectively, smaller and larger than the mean (35.2, 35.6, 30.0, 28.1, 38.1, 38.2 and 35.2% in tributaries A1, A2, FRAU1, FRAU2, SYS2, SYS3 and SYS4, respectively).

**Figure 9.**
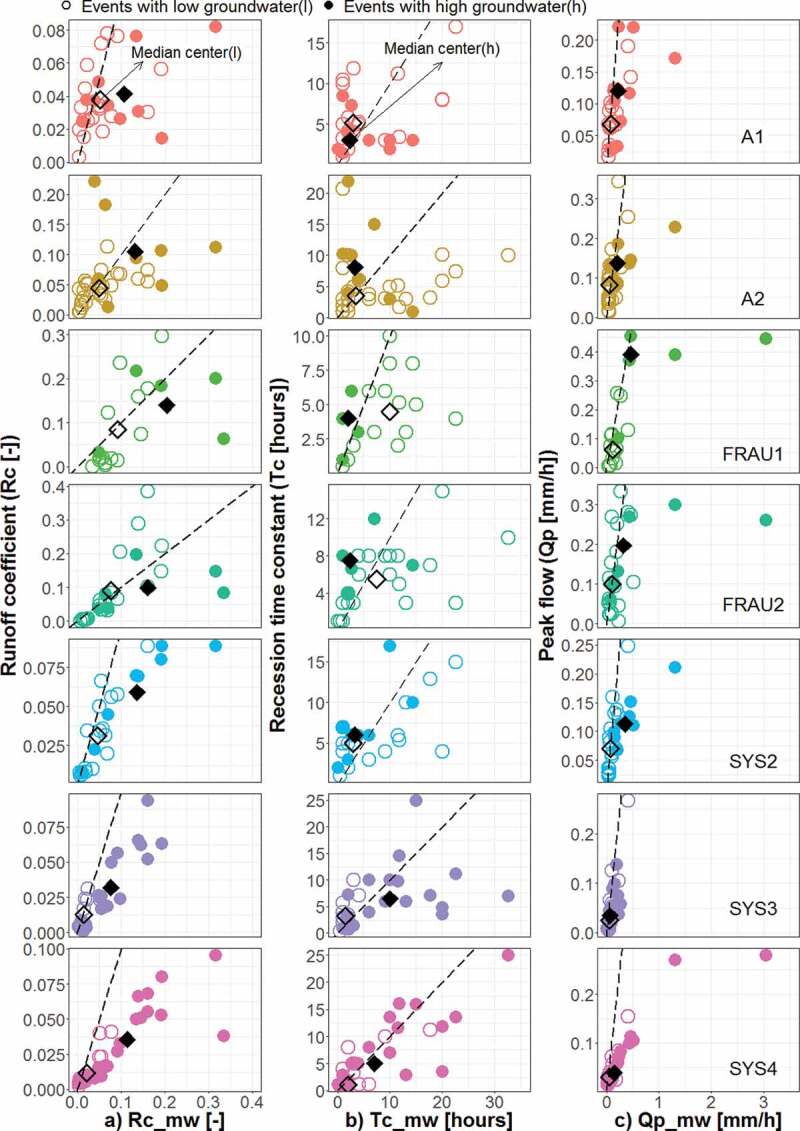
Event runoff characteristics of the tributaries plotted against those of the main outlet: (a) runoff coefficient, Rc, (b) recession time constant, Tc, and (c) peak discharge, Qp. Open and full circles indicate groundwater level prior to the runoff event, PreWL, of the tributaries that is, respectively, smaller and larger than the mean (263.44, 263.47, 263.58, 263.49, 263.45, 263.35 and 263.40 m a.s.l. in tributaries A1, A2, FRAU1, FRAU2, SYS2, SYS3 and SYS4, respectively).

Figures 6–9 plot the event runoff characteristics of the tributaries against those of the main outlet, with potential controlling factors indicated by the symbol type. Figure 6 (right panels) suggests that the peak discharges of MW are correlated with those of the tributaries as would be expected. While the small peaks of the main outlet tend to be similar to those of the tributaries, some of the large peaks are much larger than those of the tributaries (i.e. much below the 1:1 line). There are only a few events at the tile drainage systems (Frau 1, Frau2) and wetland (A2) where the peaks (in mm/h) are larger than those at MW. The runoff coefficient differs for groups classified by the mean flood peaks in different sub-catchments (Fig. 6 left panels). The median Rc at the main outlet of the events with peaks below and above the mean (0.2 mm/h) is 0.03 and 0.14, respectively. The larger Rc associated with larger peaks also occurs for the tile drainage systems (Frau1, Frau2), where the median Rc below and above the mean peak are 0.02 and 0.19 in Frau1, 0.03 and 0.15 in Frau2, respectively. In the natural drainage (Sys2, Sys3, Sys4) and the wetland (A1, A2) systems, Rc is much less related to the magnitude of runoff peaks, and the difference between the median Rc for larger and smaller peaks is very small. Table 3 shows p–values of the Kolmogorov–Smirnov two-sample test (KS) indicating differences in the group distributions, when stratifying by different characteristics according to their mean. The Rc has statistically different distributions for smaller and larger Qp only for wetlands (mainly A2), inlet pipe (Sys4) and main outlet (MW).

**Table 3.** Statistical analysis (*P* values of a two-sample Kolmogorov–Smirnov test) of the distributions of Rc, Tc and Qp for events with smaller and larger flood peaks (Qp), precipitation volumes (Pvol), initial soil moisture (PreSM) and initial groundwater levels (PreWL). The two samples are created by splitting the events according to the mean of Qp, Pvol, PreSM and PreWL of each tributary (open and full circles in Figs. 6–9). Variables with significantly different distributions at the 5% level are indicated in bold.

		Qp		Pvol			PreSM			PreWL		
Gauge	Runoff generation system	Rc	Tc	Rc	Tc	Qp	Rc	Tc	Qp	Rc	Tc	Qp
A1	Wetland	0.070	0.472	**0.009**	0.521	**0.000**	0.632	0.935	0.124	0.178	0.777	0.533
A2	Wetland	**0.015**	0.273	0.466	0.813	**0.005**	0.942	0.977	0.099	**0.002**	0.147	0.150
Frau1	Ephemeral tile drain	0.094	0.598	0.349	0.435	**0.020**	0.651	0.519	0.345	**0.018**	**0.011**	0.410
Frau2	Ephemeral tile drain	0.588	0.911	0.653	0.900	**0.037**	0.137	0.377	0.513	**0.002**	**0.006**	0.063
Sys2	Natural drainage	0.302	0.477	0.254	1.000	**0.001**	0.246	0.556	0.345	**0.001**	**0.017**	0.227
Sys3	Natural drainage (with wetland)	0.919	0.081	0.155	0.675	**0.000**	**0.001**	**0.003**	0.379	**0.037**	**0.014**	0.626
Sys4	Natural drainage (inlet pipe)	**0.012**	0.407	**0.027**	0.331	**0.000**	**0.005**	**0.000**	0.207	**0.000**	**0.000**	0.334
MW	Outlet (aggregated system)	**0.022**	0.107	0.081	0.444	**0.000**	0.081	**0.000**	0.279	**0.000**	**0.000**	0.006

Similar patterns can be observed for Tc (middle panels of Fig. 6). At the main outlet, the median Tc of the events with small and large peaks is 2 and 3 hours, respectively.

While for the natural drainage systems (Sys2, Sys3, Sys4) the events with larger peaks (i.e. larger than the mean) tend to have larger Tc at both MW and tributaries, this is not the case for the other systems. In the tile drainage systems (Frau1 and Frau2), in fact, the opposite is the case. The median Tc of smaller events (in terms of flood peak) at the tributaries corresponds to larger Tc at the MW. The larger events have larger Tc at the tile drainage tributaries, but those events tend to have smaller Tc at the MW. For the wetland systems (A1, A2), the difference between median Tc for larger or smaller peaks at MW is very small, indicating that runoff generation processes are rather disconnected. Table 3 shows that there is no large statistically significant effect on grouping of Tc according to mean Qp in HOAL.

Event precipitation volume, Pvol (Fig. 7), also has an important effect on the runoff characteristics at MW, and at the tributaries the runoff peaks are clearly stratified by precipitation volume. In the tile drainage systems (Frau1 and Frau2), and in Sys2, the differences of the peaks between the two precipitation groups are particularly large (Fig. 7, right panel) indicating a very non-linear runoff generation process triggered by precipitation. The subsurface tile drainage pipes are likely starting to be effective after reaching soil moisture state, which hence can accelerate and enhance the hillslope drainage process for larger precipitation volumes. Rc and Tc generally differ less for the two precipitation groups (Fig. 7, left and middle panel). Rc for the wetland system A1 for large precipitation events tends to be smaller than Rc at the main outlet. For a few small precipitation events, Rc can be larger, but the two groups of Rc (according to their means) are statistically different (Table 3). For Tc, a small difference is observed for the wetland systems between the two precipitation groups, suggesting that precipitation volume is not a relevant factor controlling differences in Tc. The tile drainage systems (Frau1 and Frau2) do indicate larger Rc for the high precipitation group, but Tc actually decreases with precipitation, suggesting a tendency for flashier response for high precipitation events. Sys 2 is similar to the tile drainage systems, but the other natural drainage system Sys3 shows less difference between the two precipitation groups. The KS test indicates that Rc samples split according to the Pvol mean are statistically significant at the 5% level only for A1 and Sys4 and there is no statistically significant difference for Tc in all HOAL catchments (Table 3).

Figures 8 and 9 evaluate the effect of initial soil moisture and initial groundwater levels on the runoff characteristics. Because of missing soil moisture data from January to July of 2013, several events are not included in Fig. 8 compared to Figs. 6, 7 and 9. In the wetland systems (A1, A2), the tile drainage systems (Frau1, Frau2) and at the outlet (MW), groundwater levels stratify Rc more than soil moisture. The differences in the group median of Rc (i.e. difference between open and full diamonds in Figs. 8 and 9) in the wetland, tile drainage and outlet systems are 0.02, 0.05 and 0.06, respectively, which is generally larger than the corresponding differences in the median Rc when stratifying by soil moisture. This is documented also by the results of the KS test (Table 3) where groups based on groundwater levels are statistically different in all tributaries except wetland A1, but soil moisture stratifies Rc only in the natural drainage systems (Sys3 and Sys4).

Similar results are found for Tc. In the wetland, tile drainage and main outlet systems, the differences in the group medians, when stratifying by groundwater, are larger than when stratifying by soil moisture. Interestingly, groundwater levels tend to increase Tc in the wetland systems of A2, but there is little effect of soil moisture on Tc. In the tile drainage systems (Frau1, Frau2), unfortunately, soil moisture data were not available for events with large Tc, so comparisons with soil moisture are not possible. For the natural drainage systems (Sys2, Sys3, Sys4), large soil moisture results in events with large Tc.

## Discussion and conclusions

### Spatial and seasonal variability of event runoff characteristics

The results show that the spatial variability of event runoff characteristics is related to the main runoff generation systems. We found that Rc tends to be the largest for the tile drainage systems (mean Rc = 0.09, standard deviation σ = 0.09) and the main outlet (mean Rc = 0.08, σ = 0.09), while it is smaller in the natural drainage systems (mean Rc = 0.03, σ = 0.02). This is consistent with previous assessments in small agricultural catchments (Cerdan *et al*. 2004, Blume *et al*. 2007, Tachecí *et al*. 2013). For example, Cerdan *et al*. (2004) found mean Rc and σ of 0.05 and 0.045, respectively, for 90 ha catchment in Normandy or Tachecí *et al*. (2013) found the Rc between 0.03 and 0.06 in 0.6 km^2^ catchment in the Czech Republic. The magnitude of Rc in HOAL is smaller than what was found on cropland hillslopes in central Iowa (Chen *et al*. 2019) where the median over 70 events was 0.22, or as reported in regional assessments of mesoscale catchments in Austria (the median of Rc varies between 0.18 and 0.43, Merz *et al*. 2006) or Germany (Tarasova *et al*. 2018b). The magnitude of Rc is not related to size or surface slope of sub-catchments in HOAL, which is similar as reported in previous studies of Chen *et al*. (2019) or Cerdan *et al*. (2004).

The analysis of recession time constants showed that Tc does not vary much between the systems and the difference between the largest Tc at the main outlet (mean Tc = 6.6 h, σ = 7.6 h), and the smallest Tc for the tile drainage systems (mean Tc = 4.2 h, σ = 2.5 h) is around only 2 hours. A comparison with a regional assessment of Tc and flood time scales of Gaál *et al*. (2012) shows that the natural drainage and aggregated systems compare well with hotspots of fast response catchments in terms of magnitude and seasonality of Tc. Wetland and tile drainage systems have generally lower Tc which indicates shallower flow paths and higher subsurface connectivity compared to natural drainage systems.

The largest Rc is estimated for the tile drainage systems and the main outlet in January and February and small values of Rc are found between July and October. The seasonal pattern of Rc value is in agreement with previous hillslope or regional studies (Hewlett and Hibbert 1967, Merz and Blöschl 2009, Norbiato *et al*. 2009, Rodríguez-Blanco *et al*. 2012) and corresponds to the higher contribution of rainfall to soil moisture changes and to the high evapotranspiration in July and August (Rodríguez-Blanco *et al*. 2012). A similar seasonal variability is observed for Tc for the main outlet and the natural drainage system with large Tc in January and February and small Tc in July and August. This is consistent with the seasonal dynamics of groundwater levels, which reflect functions of water-holding capacity in aquifer structure to recession (Thomas *et al*. 2015, Patnaik *et al*. 2015).

### Process controls on event runoff characteristics

The comparison of the runoff responses for different runoff generation systems indicates that groundwater levels explain the temporal variability of Rc and Tc at the main outlet. The sub-catchments with extensive cover of tile drain pipes are characterized by faster runoff response, larger runoff coefficient Rc and shorter recession time constants. The study of Silasari *et al*. (2017) shows that overland flow events do not occur frequently and, in the study area, these are generated mainly by saturation excess mechanisms and the connectivity of runoff flow paths rather than by infiltration excess processes. This is consistent with the impact of initial groundwater levels on Rc and Tc. The wetland systems tend to be disconnected from the rest of the catchment as Rc and Tc are not explained by groundwater levels or soil moisture and, apparently, by shallower drainage processes.

The results of our study show that, at a small catchment scale, event precipitation volume variability is very small and it does not have an impact on the event runoff characteristics. Event precipitation volume determines the magnitude of runoff peaks, but is not related much to the event runoff coefficients or recession time constants. Neither is precipitation intensity a big control of the variability in runoff response. Our results indicate that precipitation volume in the HOAL plays a role in predicting Rc only for the main outlet. This is in agreement with a previous study of Blume *et al*. (2007) who reported an increase in runoff coefficients with total precipitation. Blume *et al*. (2007) attributed this finding not only to the precipitation water volume routed to the stream, but also to the effect of rising groundwater tables, groundwater mounding (increasing hydraulic gradients), pipe flow, and also saturation overland flow. Our results are in line also with previous findings of Chen *et al*. (2020) who indicates that precipitation characteristics and in particular precipitation duration is a factor controlling prediction of Rc for the tile drainage and outlet systems. The weak correlation with precipitation intensity found in previous studies of Kostka and Holko (2003), Tachecí *et al*. (2013) and Chen *et al*. (2020) indicate that in small catchment variability in precipitation intensity does not control variability in runoff response. Rainfall amount and intensity are likely important only when the groundwater table is close to the surface as indicated by García-Ruíz *et al*. (2008).

Our findings indicate that, at the small catchment scale, the impact of different runoff generation systems on the variability of runoff response is significant. In the next studies, we plan to further investigate the scale where geology, climate and runoff generation system interact and have an effect on runoff response, i.e. to examine how the subsurface structure and rainfall characteristics affect spatial and temporal variability of runoff generation.

## Appendix

**Table A1.** List of events with runoff coefficient, Rc, recession time constant, Tc (h), and peak discharge, Qp (mm/h). NA: missing discharge observation because of equipment failure or regular maintenance; NI: runoff event that was not identified by the event separation procedure.

Event start Event end (dd/mm/yy hh)	MW	A1	A2	Frau1	Frau2	Sys2	Sys3	Sys4
2013–01-04 16:00:00 2013–01-09 13:00:00	Rc:0.18 Tc:12.54 Qp:0.25	NA	NA	NA	NA	NA	NA	NA
2013–01-29 11:00:00 2013–01-31 15:00:00	Rc:0.3 Tc:6 Qp:0.74	NA	NA	NA	NA	NA	NA	NA
2013–02-14 22:00:00 2013–02-23 14:00:00	Rc:0.28 Tc:30 Qp:0.05	NA	NA	NA	NA	NA	NA	NA
2013–02-28 02:00:00 2013–03-03 23:00:00	Rc:0.32 Tc:11.95 Qp:0.15	NA	NA	NA	NA	NA	NA	NA
2013–03-31 03:00:00 2013–04-05 18:00:00	Rc:0.24 Tc:4.46 Qp:0.13	NA	NA	NA	NA	NA	NA	NA
2013–04-18 18:00:00 2013–04-20 12:00:00	Rc:0.07 Tc:14.24 Qp:0.13	0.03 3 0.12	0.01 1 0.1	0.12 8 0.06	0.04 7 0.1	0.05 10 0.1	NI	NA
2013–05-11 09:00:00 2013–05-12 20:00:00	Rc:0.06 Tc:7 Qp:0.07	NI	0.18 15 0.13	0.01 3 0.01	0.08 12 0.06	NI	NI	0.02 5 0.02
2013–05-17 20:00:00 2013–05-19 07:00:00	Rc:0.04 Tc:2 Qp:0.15	0.04 3 0.11	0.22 21.9 0.14	NA	NI	0.02 3 0.09	NI	0.01 1.07 0.05
2013–05-30 17:00:00 2013–06-01 08:00:00	Rc:0.19 Tc:3.94 Qp:0.46	0.06 5 0.14	0.11 6.12 0.15	0.18 3 0.45	0.15 8 0.28	0.08 6 0.15	NI	0.05 5 0.11
2013–06-01 18:00:00 2013–06-03 03:00:00	Rc:0.13 Tc:1 Qp:0.43	0.08 8.51 0.12	0.09 10.25 0.14	0.22 4 0.37	0.2 8 0.27	0.07 7 0.13	NI	0.05 3 0.1
2013–06-03 13:00:00 2013–06-05 14:00:00	Rc:0.32 Tc:2.66 Qp:1.3	0.08 7.36 0.17	0.11 10 0.23	0.2 6 0.39	0.15 6.68 0.3	0.09 6 0.21	NI	0.1 5.09 0.27
2013–06-10 10:00:00 2013–06-12 07:00:00	Rc:0.05 Tc:2 Qp:0.21	0.05 4 0.12	0.06 4 0.19	0.03 4 0.1	0.04 4 0.13	NI	0.03 3 0.08	0.02 2 0.06
2013–06-22 08:00:00 2013–06-23 02:00:00	Rc:0.01 Tc:0.1 Qp:0.51	0.02 2 0.22	NI	NA	0.01 1 0.1	0.01 2 0.11	NI	0.01 1.21 0.1
2013–06-24 16:00:00 2013–06-25 22:00:00	Rc:0.33 Tc:1 Qp:3.04	NA	NA	0.06 1 0.44	0.08 8 0.26	NA	NA	0.04 2.77 0.28
2013–07-10 17:00:00 2013–07-11 11:00:00	Rc:0.02 Tc:1 Qp:0.23	0.04 1.46 0.22	0.05 1.13 0.34	NA	0.01 3 0.05	NI	0.004 1 0.04	0.01 1.45 0.07
2013–08-04 10:00:00 2013–08-05 13:00:00	Rc:0.01 Tc:2 Qp:0.09	0.02 2 0.1	0.04 3 0.17	NA	0.01 3 0.03	NI	0.02 1.67 0.13	0.01 1 0.07
2013–08-08 19:00:00 2013–08-10 14:00:00	Rc:0.02 Tc:4 Qp:0.06	0.03 5.28 0.06	0.05 6.09 0.1	NA	0.01 6 0.02	NI	0.03 7.13 0.07	0.01 1.2 0.04
2013–08-24 21:00:00 2013–08-26 12:00:00	Rc:0.02 Tc:1 Qp:0.1	0.04 10 0.06	0.06 20.7 0.04	NA	0.004 1 0.03	0.01 7 0.06	0.02 4 0.06	0.01 1 0.03
2013–08-28 08:00:00 2013–08-29 09:00:00	Rc:0.03 Tc:1 Qp:0.17	0.03 5 0.07	0.04 8 0.05	0.001 0.5 0.02	NI	0.01 4 0.07	NI	0.01 4 0.03
2013–09-16 13:00:00 2013–09-17 16:00:00	Rc:0.02 Tc:3 Qp:0.07	0.06 11.89 0.1	NI	NI	NI	0.03 6 0.11	0.02 10 0.03	0.01 3.36 0.02
2013–11-05 22:00:00 2013–11-08 21:00:00	Rc:0.05 Tc:20 Qp:0.04	0.07 8 0.07	0.07 6 0.08	NA	NI	NI	0.02 3.61 0.02	0.01 3.54 0.02
2013–11-10 17:00:00 2013–11-12 22:00:00	Rc:0.07 Tc:20 Qp:0.04	0.08 8.11 0.09	0.11 10.18 0.11	NA	0.03 15 0.02	0.02 4 0.09	0.02 4.9 0.03	0.02 11.88 0.02
2013–11-23 19:00:00 2013–11-26 03:00:00	Rc:0.09 Tc:11.5 Qp:0.15	0.08 11.22 0.12	0.07 5.21 0.14	0.02 2 0.06	0.07 8 0.1	0.06 6 0.13	0.06 9.84 0.1	0.03 11.68 0.04
2013–12-08 03:00:00 2013–12-11 11:00:00	Rc:0.16 Tc:32.56 Qp:0.05	NI	0.07 10 0.05	NA	0.1 10 0.06	NI	0.05 7 0.04	0.06 25 0.02
2014–04-06 03:00:00 2014–04-07 00:00:00	Rc:0.01 Tc:1 Qp:0.05	0.03 10.46 0.04	0.02 1 0.1	NA	NI	NI	0.002 1 0.01	0.01 1 0.03
2014–05-02 13:00:00 2014–05-04 10:00:00	Rc:0.01 Tc:2 Qp:0.04	0.03 5.87 0.03	0.03 10 0.02	NA	NI	NI	0.001 1 0.004	0.01 1 0.03
2014–05-16 09:00:00 2014–05-19 05:00:00	Rc:0.05 Tc:3 Qp:0.18	0.03 4 0.09	0.04 5 0.09	0.02 2 0.06	0.05 3 0.18	0.05 5 0.14	0.03 1.5 0.14	0.02 5 0.06
2014–05-27 20:00:00 2014–05-30 11:00:00	Rc:0.05 Tc:2 Qp:0.4	0.03 4.14 0.19	0.05 4.23 0.25	0.02 1 0.13	0.06 4 0.28	0.03 2 0.25	0.02 1 0.27	0.04 8 0.15
2014–06-20 00:00:00 2014–06-21 14:00:00	Rc:0.004 Tc:1 Qp:0.05	0.02 3.32 0.06	0.04 3 0.13	NA	NI	NI	0.005 2 0.02	0.005 1.16 0.03
2014–06-25 10:00:00 2014–06-25 22:00:00	Rc:0.004 Tc:1 Qp:0.04	0.003 1 0.02	0.01 1 0.05	NA	NI	NI	NI	0.003 1 0.02
2014–07-08 11:00:00 2014–07-09 08:00:00	Rc:0.01 Tc:1 Qp:0.03	NA	NA	NA	NA	NI	0.004 3 0.01	0.01 1 0.03
2014–07-11 01:00:00 2014–07-12 09:00:00	Rc:0.01 Tc:2 Qp:0.03	NA	0.02 1 0.1	NA	NA	0.01 5 0.02	0.004 2.92 0.01	0.01 1 0.02
2014–07-23 08:00:00 2014–07-23 23:00:00	Rc:0.01 Tc:2 Qp:0.02	NA	NI	NA	NA	NI	0.004 3.5 0.01	0.01 1.04 0.03
2014–08-09 16:00:00 2014–08-10 08:00:00	Rc:0.01 Tc:0.5 Qp:0.22	NA	NA	NA	0.0003 1 0.01	0.01 1 0.13	0.003 0.5 0.11	0.01 1 0.08
2014–08-20 01:00:00 2014–08-21 14:00:00	Rc:0.01 Tc:1 Qp:0.02	NA	NI	NA	NI	NI	0.01 5.71 0.01	0.01 1.06 0.02
2014–08-22 17:00:00 2014–08-24 09:00:00	Rc:0.005 Tc:1 Qp:0.04	NA	0.01 2 0.01	NA	NI	0.01 5 0.03	0.005 4 0.01	0.01 1 0.04
2014–08-30 04:00:00 2014–08-30 20:00:00	Rc:0.01 Tc:2 Qp:0.03	NA	0.01 2 0.03	NA	NA	0.01 2 0.03	0.01 7.26 0.01	0.01 1 0.03
2014–08-31 19:00:00 2014–09-05 10:00:00	Rc:0.05 Tc:17.58 Qp:0.06	NA	0.03 3.28 0.08	NI	0.03 7 0.05	0.07 12.94 0.08	0.03 7.13 0.04	0.02 11.23 0.03
2014–09-13 00:00:00 2014–09-16 11:00:00	Rc:0.08 Tc:9 Qp:0.11	0.03 3.05 0.07	0.05 3.12 0.13	0.02 6 0.03	0.08 8 0.1	0.06 4 0.16	0.05 6 0.09	0.04 10.06 0.06
2014–10-17 03:00:00 2014–10-18 14:00:00	Rc:0.02 Tc:6 Qp:0.03	NA	0.02 3 0.04	NA	NI	0.01 3 0.03	0.01 4 0.01	0.01 1.24 0.02
2014–10-22 14:00:00 2014–10-25 16:00:00	Rc:0.14 Tc:6 Qp:0.27	0.03 3 0.07	0.06 3.79 0.13	0.16 6 0.25	0.29 8 0.34	0.07 6 0.11	0.07 10 0.06	0.07 8 0.08
2014–12-06 20:00:00 2014–12-08 23:00:00	Rc:0.06 Tc:22.48 Qp:0.03	0.02 16.98 0.02	0.05 7.43 0.05	0.02 4 0.01	0.03 3 0.05	0.04 15 0.04	0.02 11.22 0.02	0.02 13.61 0.01
2014–12-19 11:00:00 2014–12-21 10:00:00	Rc:0.06 Tc:13 Qp:0.04	NI	0.03 3 0.04	0.004 3 0.004	0.06 3 0.12	0.03 10 0.04	0.02 6 0.02	0.02 3 0.03
2015–01-02 16:00:00 2015–01-06 17:00:00	Rc:0.16 Tc:11.76 Qp:0.1	0.03 3.45 0.03	0.06 1.74 0.07	0.18 5.17 0.12	0.39 5 0.27	0.09 5.43 0.06	0.09 14.67 0.05	0.07 16.07 0.04
2015–01-09 12:00:00 2015–01-12 20:00:00	Rc:0.19 Tc:10 Qp:0.19	0.01 2 0.03	0.05 3 0.09	0.3 8 0.26	0.22 6 0.25	0.09 17 0.07	0.06 10 0.05	0.08 13.57 0.06
2015–03-01 23:00:00 2015–03-05 18:00:00	Rc:0.1 Tc:10 Qp:0.08	0.03 3 0.03	0.07 5 0.08	0.24 10 0.11	0.2 8 0.15	NI	0.02 10 0.02	0.03 7 0.03
2015–04-01 02:00:00 2015–04-04 23:00:00	Rc:0.14 Tc:15 Qp:0.07	NA	NI	0.07 5 0.08	NI	NI	0.06 25 0.02	0.05 15.97 0.03
2015–04-16 23:00:00 2015–04-18 13:00:00	Rc:0.01 Tc:1 Qp:0.07	0.01 1 0.05	0.03 1 0.12	NA	0.004 2.85 0.01	0.01 4 0.05	0.01 8.62 0.03	0.004 1 0.02
2015–05-22 17:00:00 2015–05-25 04:00:00	Rc:0.07 Tc:8 Qp:0.17	0.04 4 0.12	0.04 2 0.15	0.03 4 0.03	0.14 4 0.13	0.04 9 0.1	0.01 4.4 0.04	0.04 8 0.11
2015–06-08 12:00:00 2015–06-08 20:00:00	Rc:0.005 Tc:1 Qp:0.04	NA	0.01 1 0.06	NA	NI	NI	NA	0.01 1.26 0.03
2015–07-08 13:00:00 2015–07-09 22:00:00	Rc:0.01 Tc:1 Qp:0.02	NA	NA	NA	NI	NI	0.005 2 0.01	0.01 1.66 0.01
2015–07-19 13:00:00 2015–07-20 12:00:00	Rc:0.004 Tc:1 Qp:0.03	NA	NA	NA	NI	NI	0.002 3.04 0.01	0.003 1.13 0.01
2015–08-16 18:00:00 2015–08-18 10:00:00	Rc:0.01 Tc:1 Qp:0.06	NA	0.01 0.5 0.06	NA	NI	0.01 2 0.05	0.004 1.5 0.03	0.01 1 0.05
2015–09-03 13:00:00 2015–09-04 12:00:00	Rc:0.01 Tc:1 Qp:0.04	NA	NA	NA	NA	0.01 3 0.03	NA	0.01 1 0.04
2015–10-07 20:00:00 2015–10-09 18:00:00	Rc:0.01 Tc:1 Qp:0.04	NA	NI	NA	0.003 3 0.003	0.02 3 0.05	NI	0.01 1 0.03
2015–10-19 08:00:00 2015–10-22 17:00:00	Rc:0.03 Tc:9 Qp:0.04	NA	NA	0.01 3 0.01	0.03 3 0.08	0.03 5 0.05	0.01 1 0.02	0.01 5 0.02
2015–11-20 07:00:00 2015–11-22 10:00:00	Rc:0.03 Tc:15 Qp:0.02	NA	NA	NA	NI	0.02 4 0.03	0.004 2 0.01	0.005 1 0.01
